# Evaluation of 3D-Printed Dry Electrodes for Surface Electromyography in Dynamic Muscle Assessment

**DOI:** 10.3390/mi17050504

**Published:** 2026-04-22

**Authors:** Ahmad O. Alokaily, Ahmed A. Aldohbeyb, Mohamed A. Almadi, Fahed K. Alnawfal, Shahad N. Alshamlan, Suhail S. Alshahrani, Khalid Alhussaini, Alaa M. Albishi, Khalid I. Aloraini, Ahmad Zahid Rao, Ziyad Aloqalaa

**Affiliations:** 1 Department of Biomedical Technology, College of Applied Medical Sciences, King Saud University, P.O. Box 10219, Riyadh 11433, Saudi Arabia; mohalmadi@ksu.edu.sa (M.A.A.); bmt.fahed@gmail.com (F.K.A.); suhailsalem@ksu.edu.sa (S.S.A.); kalhussaini@ksu.edu.sa (K.A.); kaloraini@ksu.edu.sa (K.I.A.); zaloqalaa@ksu.edu.sa (Z.A.); 2 Department of Rehabilitation Health Sciences, College of Applied Medical Sciences, King Saud University, P.O. Box 10219, Riyadh 11433, Saudi Arabia; aalbeshi@ksu.edu.sa; 3 Department of Physical Medicine and Rehabilitation, McGovern Medical School, University of Texas Health Science Center at Houston, Houston, TX 77030, USA; ahmad.z.rao@uth.tmc.edu

**Keywords:** 3D-printed electrodes, additive manufacturing, signal quality, conductive filaments, surface electromyography (sEMG)

## Abstract

Surface electromyography (sEMG) is widely used to assess muscle activity in clinical and research settings. However, while conventional wet electrodes have advanced considerably in recent years, they are often limited by disposability, reduced comfort, and limited reusability. Recent advances in additive manufacturing provide opportunities to fabricate customizable, low-cost dry electrodes using conductive filaments. This study aimed to evaluate the feasibility and signal performance of in-house-fabricated 3D-printed sEMG electrodes made from three commercially available conductive filaments, (Fili, Filaflex, and Proto-Pasta) differing in base polymer and resistivity, and compared their performance with standard wet electrodes. Surface electrodes were placed over the biceps brachii muscle, and EMG signals were recorded during concentric–eccentric elbow flexion under three loading conditions (3, 5, and 7 kg). Signal quality was assessed using EMG amplitude, signal-to-noise ratio (SNR), and background noise. The results showed no significant differences in SNR or background noise between the 3D-printed electrodes and standard wet electrodes. Among the tested materials, Proto-Pasta electrodes produced the highest mean EMG amplitudes, while Filaflex electrodes showed slightly lower background noise, although these differences were not statistically significant. Overall, the findings indicate that in-house-fabricated 3D-printed electrodes can provide signal quality comparable to conventional wet electrodes, supporting their potential use as low-cost and customizable alternatives for sEMG applications in research and wearable monitoring systems.

## 1. Introduction

Electromyography (EMG) is a widely utilized noninvasive method for evaluating the electrical activity of skeletal muscles. By detecting voltage fluctuations on the skin surface corresponding to muscle fiber depolarization and repolarization, EMG enables comprehensive analysis of motor unit recruitment, neuromuscular coordination, and muscle fatigue. Clinically, EMG plays a crucial role in diagnosing neuromuscular disorders such as amyotrophic lateral sclerosis (ALS), muscular dystrophy, and peripheral neuropathies [1]. Its applications extend to rehabilitation monitoring, prosthetic control, performance optimization in sports, and the development of human–machine interfaces [2,3].

Surface electromyography (sEMG) utilizes electrodes affixed to the skin to detect low-amplitude biopotentials. Due to this low amplitude, electrodes must possess low impedance and high sensitivity to preserve signal integrity. The prevailing standard in both clinical and research settings is the wet silver/silver chloride (Ag/AgCl) electrode, which comprises a silver chloride-coated sensor enveloped by a conductive gel. This gel establishes an ionic interface with the skin, thereby reducing impedance and facilitating efficient ionic-to-electronic current transduction [4]. Under controlled conditions, wet electrodes provide high-fidelity recordings with minimal baseline noise. Despite these advantages, wet electrodes are suboptimal for long-term or wearable use. The conductive gel tends to dry over time, increasing impedance and diminishing signal quality. Their application often requires skin preparation, which can cause irritation or allergic reactions when used for extended periods. Furthermore, wet electrodes are generally disposable, which contributes to elevated costs and environmental concerns [4].

Such limitations have driven substantial efforts to develop alternatives to conventional wet electrodes such as Ag/AgCl electrodes. Liquid metal-based, textile-integrated, and other stretchable dry electrodes offer excellent conformability, low impedance, and long-term stability for ECG, EMG, and EEG monitoring [5,6]. Liquid and semi-liquid metal systems, for instance, enable highly stretchable, conformal electrodes with low skin–electrode impedance suitable for high-resolution recordings [6,7]. Similarly, gold- and gold–graphene-based dry electrodes have shown low impedance, high sensitivity, and robust performance in biopotential recording, in some cases matching or surpassing Ag/AgCl electrodes [8,9,10]. However, many of these technologies rely on complex designs and specialized fabrication processes, limiting their accessibility to a broader user base. In contrast, additive manufacturing enables electrode designs that are customizable, affordable, and scalable. Recent advances in materials science further support this approach by providing printable electrode materials and architectures tailored to diverse application requirements.

Recent advancements in additive manufacturing have opened up new possibilities for designing dry sEMG electrodes with customizable geometries, affordability, and scalability. Three-dimensional (3D) printing enables rapid prototyping of electrode structures tailored to individual anatomies, with functional surface textures and geometries that enhance skin contact. Conductive thermoplastic filaments, such as polylactic acid (PLA) and thermoplastic polyurethane (TPU), offer a combination of mechanical flexibility and electrical conductivity, making them appealing for biopotential sensing [11].

The feasibility of 3D-printed electrodes for electrophysiological recording has been demonstrated across several modalities, particularly electrocardiography (ECG) and electroencephalography (EEG). Hybrid fabrication approaches that combine 3D-printed substrates with electroless metal plating have produced EMG and ECG electrodes with impedance values approaching clinical standards (~66 kΩ at 50 Hz), although this comes at the cost of increased fabrication complexity [12]. Fully 3D-printed dry EEG electrodes fabricated from conductive filaments have also achieved low impedance and reliable alpha-wave detection, indicating that entirely printed structures can support effective signal acquisition without additional metallization [11]. In sEMG, DMLS-printed microneedle dry electrodes have preserved signal fidelity under static conditions [9,13], whereas carbon black-doped TPU electrodes fabricated directly using consumer-grade multi-material FDM printers have produced EMG amplitudes comparable to Ag/AgCl electrodes and have been integrated into multichannel gesture-recognition bands, highlighting the promise of low-cost systems for dynamic applications [14]. Comparisons between silver-ink-based and carbon black–TPU 3D-printed sEMG electrodes further indicate that silver-ink traces can substantially reduce electrode–skin impedance and improve signal stability during muscle contractions [15].

Electrode geometry and mechanical design are also critical determinants of signal quality. Serpentine conformal 3D-printed electrodes integrated into wearable wristbands have been shown to enhance skin contact and reduce motion artifacts, while printed conductive traces and electrodes on flexible substrates have demonstrated viability for EMG and other biopotential systems under movement [16]. Stretchable high-density EMG arrays on flexible printed circuit boards have achieved performance comparable to, or exceeding, conventional EMG grids in high-motion environments, underscoring the importance of dense, mechanically compliant architectures [17]. Parallel material developments have shifted toward softer, more conformal biointerfaces, including 3D-printed hydrogel arrays and other soft conductive networks that maintain low impedance and stable long-term sEMG signals suitable for real-time prosthetic control [18]. Furthermore, softer and more compliant interfaces have expanded the material landscape [19]. For example, supersoft, ultra-stretchable PEDOT:PSS-based hydrogel electrodes have maintained durable skin contact and reduced motion artifacts, underscoring the value of mechanically compliant biointerfaces for stable electrophysiological sensing [20]. Recent reviews further indicate that additive manufacturing is emerging as a versatile platform for dry electrophysiological electrodes, offering improved comfort, conformability, biocompatibility, and design flexibility while continuing to address persistent challenges such as electrode–skin impedance and mechanical durability at small scales [21]. These findings indicate that additive manufacturing can produce customizable, low-cost, and reusable biosignal interfaces; however, most studies have focused on ECG, resting-state measurements, or custom materials, and direct quantitative evaluation of off-the-shelf conductive filaments for EMG acquisition during dynamic contractions remains limited.

Although additive manufacturing has shown considerable potential for producing customizable, low-cost, and reusable biosignal interfaces, most existing systems have been evaluated primarily in cardiac monitoring, resting-state EEG/EMG, or custom-engineered material platforms [22,23,24]. Relatively few studies have examined the use of off-the-shelf conductive filaments for sEMG acquisition during dynamic muscle contractions, where concentric and eccentric movements introduce substantial motion artifacts and fluctuations in skin–electrode contact. Consequently, the extent to which directly 3D-printed dry electrodes fabricated from commercially available conductive PLA filaments can quantitatively match conventional wet Ag/AgCl electrodes for dynamic sEMG remains unclear.

In this study, we assessed the performance of three 3D-printed electrodes, fabricated using commercially available conductive filaments (Fili, Filaflex, and Proto-Pasta), in capturing surface electromyography (sEMG) signals from the biceps brachii during dynamic elbow flexion–extension under increasing load. Standard Ag/AgCl electrodes served as the reference. By comparing these electrodes under physiologically relevant load conditions, we aimed to determine whether low-cost, readily manufacturable, in-house-fabricated dry electrodes could provide a viable alternative to wet electrodes for dynamic sEMG acquisition. This approach holds potential for cost-effective and scalable applications in both clinical diagnostics and research laboratories, particularly in resource-constrained or wearable system development settings.

## 2. Materials and Methods

### 2.1. Participants

Eighteen healthy young adults (17 males and 1 female; mean age = 25.17 ± 1.42 years) participated in this study. All participants reported no history of neuromuscular disorders and were free of injuries that could affect muscle performance. Informed consent was obtained from all participants. This study was approved by the Institutional Review Board of King Saud University (E-25-9856).

### 2.2. Electrode Materials, Design, and 3D Printing

Three commercially available conductive filaments, Proto-Pasta, Filaflex, and Fili, were used to fabricate the 3D-printed electrodes evaluated in this study. For comparison, a standard pre-gelled Ag/AgCl adhesive electrode (14 × 34 mm; Spes Medica, Genova, Italy) was used as a reference.

Proto-Pasta is a widely used conductive filament composed of polylactic acid (PLA) doped with carbon black, exhibiting a volume resistivity between 4.8 and 8.4 Ω·cm [25]. Filaflex is a thermoplastic polyurethane (TPU)-based flexible filament, and its conductive variant incorporates conductive additives to enable electrical conductivity [26]. In this study, the conductive version of Filaflex was used. Fili, developed by AIMPLAS, is also a TPU-based composite with a reported volume resistivity of 27.44 Ω·cm [27], representing a flexible material with comparatively higher resistivity. The inclusion of both PLA-based and TPU-based filaments enables evaluation of the trade-off between electrical conductivity and mechanical flexibility, which is critical for maintaining stable electrode–skin contact during dynamic conditions.

**Figure 1:**
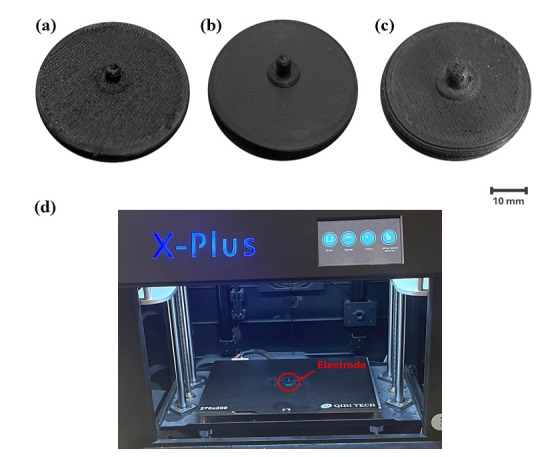
3D-printed electrodes and fabrication setup. Electrodes made from (**a**) Filaflex, (**b**) Proto-Pasta, and (**c**) Fili, and (**d**) 3D printer (Qidi Tech X-Plus) used for fabrication.

All filaments were used to fabricate circular electrodes with a diameter of 40 mm and a thickness of 5.5 mm, incorporating a central knob structure (Figure 1). The knob had an inner diameter of 4 mm, an outer diameter of 9 mm, and a thickness of approximately 3.5 mm [23]. This geometry was selected to provide sufficient contact area for signal acquisition while enhancing localized pressure at the center of the electrode, thereby improving electrode–skin contact stability during movement and reducing motion artifacts.

The electrodes were fabricated using a Qidi Tech X-Plus fused deposition modeling (FDM) 3D printer (Qidi Technology Co., Ltd., Wenzhou, China). The printing parameters for each filament are summarized in Table 1. These parameters were selected based on filament manufacturer recommendations and the default settings of the QIDI Slicer software (version 1.1.4) to ensure reproducible printing conditions. The infill density (20%), layer height (0.2 mm), and cooling fan speed were kept constant across all materials. No post-processing treatments, such as conductive coating or surface modification, were applied. This approach was adopted to evaluate the intrinsic performance of the filaments in their as-printed state and to emphasize the feasibility of rapid, low-cost fabrication without additional processing steps.

### 2.3. sEMG Recording

Surface EMG signals were recorded using a clinical-grade Surpass LT system (EMS Biomedical, Korneuburg, Austria). Bipolar surface electrodes were placed on the biceps brachii muscle. The sEMG signals were digitized at 60 kHz and stored for offline analysis.

### 2.4. Experimental Protocol

The participants were seated comfortably in an armchair, with their elbows and forearms supported on the armrests. The skin over the right biceps brachii was prepared according to standard surface EMG procedures, and both the skin and 3D-printed electrodes were cleaned with an alcohol swab. Bipolar surface electrodes were positioned over the biceps brachii muscle, and a ground electrode was placed on the wrist. The standard Ag/AgCl electrodes were self-adhesive, whereas the 3D-printed electrodes were secured using rubber bands.

Participants initially performed isometric arm flexion without any external load to verify signal quality and ensure proper electrode placement. Upon confirmation, the load test began. Each participant completed trials with all three weights (3, 5, and 7 kg) in a randomized and counterbalanced order. For each load, the participants performed repeated elbow flexion–extension cycles by lifting the weight toward the shoulder (concentric contraction), followed by a controlled lowering (eccentric contraction) while keeping the elbow supported on the armrest. Each contraction cycle was followed by an approximately 7-s rest period. The lift–rest sequence was repeated four times for each electrode type. The order of electrode placement and sequence of load conditions were randomized and counterbalanced across participants.

### 2.5. sEMG Signal Processing and Evaluation

Digital signal processing was performed using MATLAB R2023b (MathWorks, Natick, MA, USA). EMG signal processing followed procedures consistent with those reported in previous studies [13,14]. Briefly, the raw EMG signals were band-pass-filtered between 20 Hz and 250 Hz using a zero-phase 3rd-order Infinite Impulse Response (IIR) filter. A notch filter at 60 Hz was used to eliminate powerline interference. The filtered sEMG signals were rectified and smoothed using a 20 ms moving root-mean-square (RMS) window to extract the signal envelope. The RMS was calculated as shown in Equation (1):

(1) $$RMS=\sqrt{\frac{1}{N}\sum_{n=1}^{N}x_{n}^{2}}$$

where $x_{n}$ is the band-pass-filtered sEMG signal and *N* is the window length in samples. The envelope signal was subsequently segmented to isolate the individual muscle contractions (4 repetitions). For comparison across electrode types, several metrics were extracted from each trial: SNR, maximum amplitude, and peak-to-peak noise amplitude. The peak-to-peak background noise was measured as the difference between the maximum and minimum values of the RMS envelope during the resting period. For SNR calculation, the signal segment was defined as the time window corresponding to active muscle contraction, identified from the RMS envelope, while the noise segment was defined as the baseline recording at rest. The SNR was calculated using the *snr* function in MATLAB, which computes it as the ratio of the RMS of the signal values of the sEMG signal during contraction to the RMS of the background (Equation (2)).

(2) $$SNR=20\log_{10}\left(\frac{RMS_{contraction}}{RMS_{background}}\right)$$

### 2.6. Statistical Analysis

Statistical analysis was performed to determine whether electrode type and load level had significant effects on EMG-derived parameters: SNR, mean maximum EMG amplitude, and background noise. A two-way repeated-measures ANOVA was conducted separately for SNR and mean maximum amplitude, with electrode type and load as within-subject factors. Post hoc comparisons were performed using paired sample *t*-tests. Additionally, a one-way repeated-measures ANOVA was used to compare the peak-to-peak background noise values across different electrode types under resting conditions. Statistical analyses were performed using IBM SPSS Statistics (version 30.0; Armonk, NY, USA: IBM Corp.). Results are reported as mean ± standard deviation (SD), and statistical significance was set at *p* < 0.05.

## 3. Results

**Figure 2:**
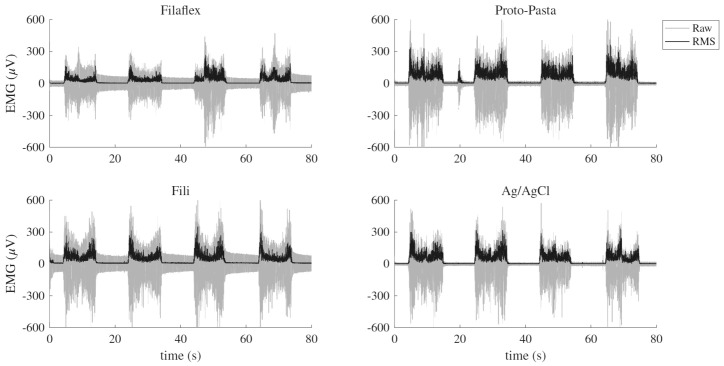
A representative EMG recording acquired using four different electrode types (Filaflex, Proto-Pasta, Fili, and Ag/AgCl) during repeated concentric–eccentric arm flexion tasks. The gray trace represents the raw EMG signal, and the black trace shows the RMS envelope used for signal amplitude estimation.

All participants completed the experimental protocol, and all acquired EMG signals were included in the analysis. Each electrode type produced usable sEMG recordings with observable muscle activation at all load levels (Figure 2).

### 3.1. Signal-to-Noise Ratio (SNR)

**Figure 3:**
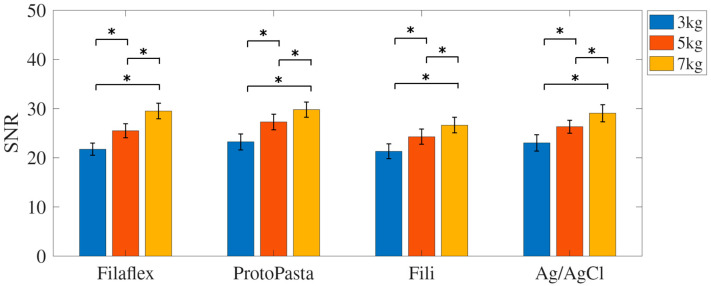
Mean SNR (dB) for each electrode type (Filaflex, Fili, Proto-Pasta, and Ag/AgCl) under three loading conditions (3, 5, and 7 kg). A statistically significant increase in the SNR was observed with increasing load across all electrode types (*p* < 0.05), whereas no significant differences were found between the electrode materials. The results are shown as mean ± standard error. * denotes *p* < 0.05.

Descriptive analysis indicated a consistent increase in the SNR with increasing load across all electrode types. For the Fili electrode, the SNR values were 21.31 ± 6.31 at 3 kg, 24.28 ± 6.58 at 5 kg, and 26.66 ± 6.72 at 7 kg. The Proto-Pasta electrode recorded values of 23.25 ± 6.90 at 3 kg, 27.29 ± 6.70 at 5 kg, and 29.79 ± 6.59 at 7 kg. The Filaflex electrode showed SNR values of 21.73 ± 5.23 at 3 kg, 25.50 ± 6.16 at 5 kg, and 29.51 ± 6.66 at 7 kg. For the standard Ag/AgCl electrode, values were 23.03 ± 6.96 at 3 kg, 26.33 ± 5.64 at 5 kg, and 29.08 ± 7.30 at 7 kg (Figure 3).

A two-way repeated-measures ANOVA revealed a statistically significant main effect of load on the SNR (F(2,34) = 73.19, *p* < 0.001, partial η^2^ = 0.81), indicating a substantial increase in the SNR with heavier loads. The main effect of electrode type was not statistically significant (F(3,51) = 1.02, *p* = 0.39), and there was no significant interaction effect between electrode type and load on SNR (F(6,102) = 1.27, *p* = 0.29).

Paired-sample *t*-tests showed that the differences in SNR between each pair of load levels (3 kg vs. 5 kg, 5 kg vs. 7 kg, and 3 kg vs. 7 kg) were statistically significant for all electrode types (*p* < 0.01), indicating a significant increase in SNR with load across all electrode types.

### 3.2. EMG Amplitude

**Figure 4:**
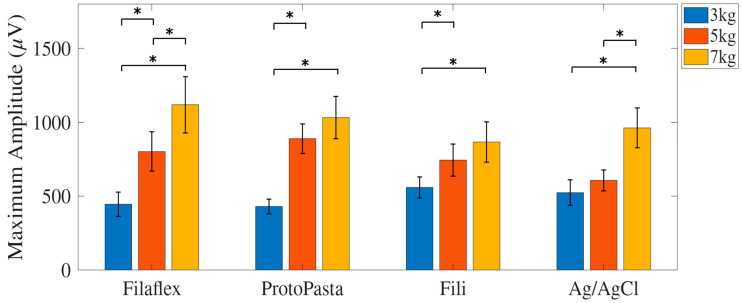
Average peak RMS amplitude across participants (µV) recorded across all electrode types (Filaflex, Fili, Proto-Pasta, and Ag/AgCl) under three loading conditions (3, 5, and 7 kg). A statistically significant increase in EMG amplitude was observed with increasing load for all electrode types (*p* < 0.05), with no significant main effect observed for the electrode type. The results are shown as mean ± standard error. * denotes *p* < 0.05.

The maximum EMG RMS amplitude increased systematically with increasing load across all electrode types. For the Fili electrode, the mean amplitudes were 430.51 ± 214.04 µV at 3 kg, 744.38 ± 464.41 µV at 5 kg, and 866.88 ± 577.50 µV at 7 kg. The Proto-Pasta electrode yielded average amplitudes of 559.06 ± 301.06 µV, 889.83 ± 425.43 µV, and 1031.93 ± 605.59 µV at the respective loads. Filaflex recordings showed values of 445.59 ± 351.24 µV at 3 kg, 801.97 ± 567.29 µV at 5 kg, and 1118.95 ± 807.21 µV at 7 kg. The Ag/AgCl electrode produced amplitudes of 530.59 ± 365.73 µV, 607.03 ± 299.8 µV, and 962.11 ± 570.56 µV under the same weight conditions (Figure 4).

A two-way repeated-measures ANOVA revealed a significant main effect of load on EMG amplitude (F(2,34) = 22.92, *p* < 0.001, partial η^2^ = 0.57), whereas the main effect of electrode type was not statistically significant (F(3,51) = 1.31, *p* = 0.28). No significant interaction between electrode type and load was observed.

Post hoc paired-sample *t*-tests revealed condition-dependent differences in amplitude across load levels within each electrode type. For the Fili electrodes, amplitude increased significantly between 3 and 5 kg (*p* = 0.003) and between 3 and 7 kg (*p* < 0.001), while the difference between 5 and 7 kg was not statistically significant (*p* = 0.14). For the Proto-Pasta electrodes, a significant increase was observed between 3 and 5 kg (*p* = 0.01) and between 3 and 7 kg (*p* = 0.002), with no significant difference between 5 and 7 kg (*p* = 0.34). The Filaflex electrodes showed significant differences across all pairwise comparisons, including 3 and 5 kg (*p* = 0.001), 3 and 7 kg (*p* < 0.001), and 5 and 7 kg (*p* = 0.008). For the Ag/AgCl electrodes, there was a significant difference between 3 and 7 kg (*p* = 0.003) and between 5 and 7 kg (*p* = 0.004), while the comparison between 3 and 5 kg was not statistically significant (*p* = 0.34).

### 3.3. Background Noise

**Figure 5:**
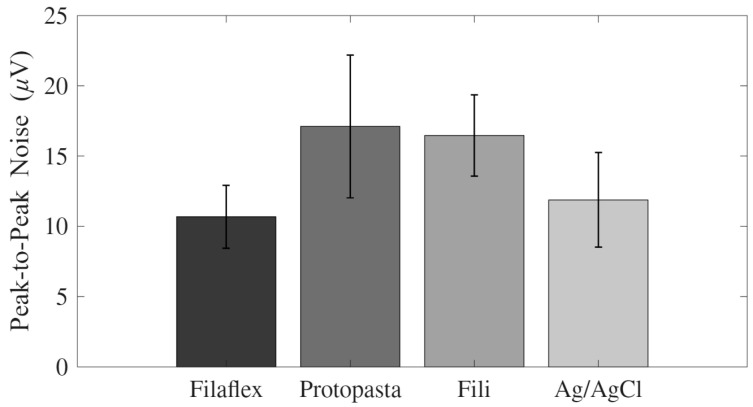
Mean peak-to-peak background noise (µV) recorded under resting conditions for each electrode type (Filaflex, Proto-Pasta, Fili, and Ag/AgCl). No statistically significant differences in background noise were observed between the electrode types (*p* > 0.05). The results are shown as mean ± standard error.

The peak-to-peak RMS background noise was measured under resting conditions for each electrode type. The mean noise level for the Fili electrode was 16.46 ± 12.24 µV, whereas that of Proto-Pasta was 17.11 ± 21.57 µV. The Filaflex electrode produced a lower mean noise value of 10.68 ± 9.46 µV, while the standard Ag/AgCl electrode had a mean of 11.87 ± 14.27 µV (Figure 5).

A one-way repeated-measures ANOVA revealed no significant effect of electrode type on the RMS background noise (F(3,51) = 0.93, *p* = 0.44, partial η^2^ = 0.05). The background noise levels did not differ significantly among the four electrode types at rest.

## 4. Discussion

This study evaluated the performance of commercially available 3D-printed dry electrodes for sEMG acquisition compared with conventional Ag/AgCl wet electrodes. All tested electrodes (Fili, Filaflex, and Proto-Pasta) were able to detect EMG signals during dynamic contractions, with no statistically significant differences observed in SNR, maximum amplitude, or background noise. Importantly, the increase in SNR and EMG amplitude with higher loads was consistent across electrode types, reflecting the physiological principle of progressive motor unit recruitment during increased muscular effort [28].

The post hoc analysis further revealed differences in how the electrodes detected increases in EMG amplitude with increasing load levels. Filaflex electrodes exhibited significant differences across all load comparisons, indicating a consistent graded response to increasing contraction intensity. In contrast, Fili and Proto-Pasta electrodes did not distinguish between intermediate and high loads, suggesting reduced sensitivity at higher contraction levels. Similarly, Ag/AgCl electrodes primarily differentiated between the lowest and highest loads, reflecting a more threshold-like response. These findings suggest that, while overall signal amplitude and SNR were comparable across electrode types, the ability to resolve finer changes in muscle activation may vary between electrodes. These findings extend prior work that has predominantly focused on ECG or static EMG conditions. Previous studies have highlighted the limitations of dry electrodes under dynamic conditions [4], while high SNR has been reported for Proto-Pasta electrodes in ECG monitoring [22]. By evaluating electrode performance during concentric–eccentric contractions, which are more susceptible to motion artifacts, the present study demonstrates that 3D-printed dry electrodes can maintain stable signal acquisition under dynamic conditions. Low-impedance performance has also been achieved using more complex fabrication approaches, such as electroless copper/gold plating [12], whereas conductive filament-based electrodes have been shown to support effective EEG acquisition without additional surface modification [11]. In this context, the present findings suggest that commercially available conductive filaments, even without specialized materials or post-processing, are capable of providing sEMG performance comparable to conventional wet electrodes, thereby supporting their use as low-cost and scalable alternatives.

Electrode material and geometry are important contributors to signal quality. In this study, Filaflex consistently exhibited lower background noise, while Proto-Pasta produced the highest mean amplitudes, though neither difference reached statistical significance. The flexibility of Filaflex may enhance skin conformity and reduce motion artifacts, consistent with prior work on soft and conformal electrodes [14,17].

Despite the encouraging results, several limitations should be acknowledged. First, this study focused on the biceps brachii muscle under controlled laboratory conditions, which may not reflect electrode performance during ambulatory or high-motion activities. Second, the participant cohort was predominantly male (17 males and 1 female), representing a significant sex imbalance. Differences in skin properties, including subcutaneous fat distribution, skin hydration, and hair density, may influence electrode–skin impedance and sEMG signal quality, limiting the generalizability of the findings across sexes. In addition, several important performance parameters were not directly evaluated, including electrode–skin impedance, impedance variation under mechanical deformation, stretchability, and mechanical compliance under strain. Furthermore, durability and long term reusability under several conditions were not assessed. The study also relied on a clinical grade acquisition system with high sampling rates, which may not fully represent performance in low power wearable systems. Future work should address these limitations by evaluating electrode performance under real world wearable conditions, incorporating more diverse participant populations, and systematically investigating the mechanical and electrical behavior of conductive filaments under deformation.

In conclusion, this study demonstrates that dry electrodes fabricated from commercially available conductive filaments can achieve performance comparable to standard Ag/AgCl electrodes for dynamic sEMG acquisition. However, differences in sensitivity to incremental load changes indicate that material selection may influence the resolution of muscle activation patterns. These findings support the feasibility of low-cost, additively manufactured electrodes for research, wearable systems, and potential clinical applications.

## Figures and Tables

**Table 1:** Printing parameters for 3D-printed electrode.

Print Settings/Electrode	Proto-Pasta	FilaFlex	Fili
Nozzle Temperature (°C)	215	245	250
Print Speed (mm/s)	45	20	40
Layer Height (mm)	0.2	0.2	0.2
Infill Density (%)	20	20	20
Build Plate Temperature (°C)	60	60	60
Bed Adhesion Method	PEI + Glue Stick	PEI + Glue Stick	PEI + Glue Stick
Cooling Fan Speed	100%	100%	100%

## Data Availability

The data that support the findings of this study are available from the corresponding authors upon reasonable request.
